# Electrodeposition of Cu nanoparticles on TiO_2_ nanotubes for enhanced bactericidal effect against *Escherichia coli*

**DOI:** 10.1039/d5ra07012k

**Published:** 2025-11-13

**Authors:** Naser Edraki, Elnaz Moslehifard, AmirAli Farmani, Ali Baradar Khoshfetrat, Farzad Nasirpouri

**Affiliations:** a Faculty of Materials Engineering, Sahand University of Technology Tabriz 51335-1996 Iran nasirpouri@sut.ac.ir; b Advanced Materials Research Institute, Faculty of Materials Engineering, Sahand University of Technology Tabriz 5331817634 Iran; c Department of Prosthodontics, Faculty of Dentistry, Tabriz University of Medical Sciences Tabriz Iran; d Faculty of Chemical Engineering, Sahand University of Technology Tabriz 51335-1996 Iran

## Abstract

Effective and environmentally friendly disinfection methods are crucial for preventing infections and deaths. In this work, we produced anodic TiO_2_ nanotubes (TNTs) composited with copper nanoparticles *via* electrodeposition. The TNTs were prepared by two-step anodic oxidation, after which Cu nanoparticles were deposited onto them using cyclic voltammetry (CV) for 2, 3, and 5 cycles at scan rates of 20, 50, and 100 mV s^−1^. Field emission scanning electron microscopy (FESEM) images revealed that deposition at the highest scan rate (100 mV s^−1^) achieved superior nanoparticle dispersion on the TNTs compared to lower rates. Diffuse reflectance spectroscopy (DRS) and Mott–Schottky analysis indicated that the sample prepared with 2 cycles at 100 mV s^−1^ exhibited the best photocatalytic activity. For this optimal composite, the band gap and flat band potential were 1.68 eV and −0.579 V, respectively, compared to 3.32 eV and −0.631 V for pristine TNTs. The bactericidal activity of the TNTs and Cu-deposited TNTs was tested against *Escherichia coli* (*E. coli*) under visible light irradiation. The composite electrodeposited with 2 cycles at 100 mV s^−1^ exhibited bactericidal activity approximately 79.3% higher than that of pristine TNTs under visible light and 28.2% higher in the dark. This study demonstrates a cost-effective and efficient method for fabricating composites with high bactericidal performance for medical and dental applications.

## Abstract 2

Effective and environmentally friendly disinfection methods are crucial for preventing infections and deaths. In this work, we produced anodic TiO_2_ nanotubes (TNTs) composited with copper nanoparticles *via* electrodeposition.

## Graphical abstract

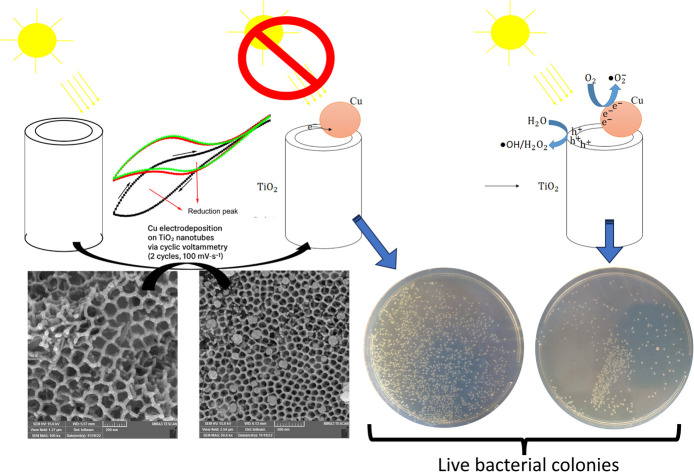

## 1. Introduction

The global presence of microbes and the diseases they cause pose a significant problem for modern society. While some microbes are beneficial, many are dangerous to humans, animals, and plants.^1^ There are many disinfectants available worldwide, among which chemical disinfectants are some of the most important. Although these chemical disinfectants are very effective against many microbes, they are often volatile and can be toxic or carcinogenic to humans. Recently, photocatalytic disinfectants have been studied as alternatives due to their safety for humans and the environment.^2–4^ Nanoparticles have many applications in various fields, including electrical, mechanical, and medical, due to their large specific surface area.^5,6^ Some metal oxide nanostructures (*e.g.*, TiO_2_, Al_2_O_3_, CuO, and ZnO) are used as antibacterial materials. Their bactericidal activity depends on chemical composition, shape, size, and environmental conditions.^7,8^ Similarly, the most studied metal oxide nanoparticles—ZnO, TiO_2_, Ag_2_O, and CuO—are selected for their proven high antibacterial activity and biocompatibility.^9,10^ Meanwhile, zirconia (ZrO_2_) nanoparticles are widely used as additives to enhance material properties like mechanical strength.^11^

Among the wide application of TiO_2_, we can mention water splitting,^12^ disinfection^13^ and self-cleaning.^14^ This material exists in three crystalline phases: brookite, anatase, and rutile. Among these, the anatase and rutile phases are more stable than brookite.^15^ Anatase TiO_2_ is one of the most important materials in the field of photocatalysis due to its high stability and strong oxidizing power.^16,17^ TiO_2_ nanostructures exhibit excellent disinfection properties due to their photocatalytic activity, which induces toxicity in bacteria under UV light irradiation.^18^ First in 1985, Matsunaga *et al.*^19^ reported bactericidal activity of TiO_2_–Pt powder under UV light. Later many researches were performed in the field of bactericidal activity of TiO_2_ base materials. Non-deposited TiO_2_ has a band gap energy of 3.2 eV, which restricts its photocatalytic activity to UV light; however, UV light constitutes less than 5% of the solar spectrum.^20^ According to previous reports, the addition of some metals (*e.g.*, copper,^21–23^ silver,^24^ gold,^25^ iron^26^ and selenium^27^) on TiO_2_ substrate, can make it sensitive to visible light. This is due to a shift of the absorption edge into the visible light region and a decrease in band gap energy. These changes facilitate electron transfer from the valence band to the conduction band. The increased electron transfer enhances photocatalytic reactions, thereby intensifying the photocatalytic disinfection process.

Numerous studies have investigated the bactericidal activity of TiO_2_–Cu nanostructures. The addition of copper to the TiO_2_ substrate creates intermediate energy bands and suppresses the recombination of electron–hole pairs.^23^ The primary mechanism of photocatalytic disinfection in these materials is the production of reactive oxygen species (ROS), which is driven by photocatalytic reactions within the nanostructures.^28,29^ Also these nanostructure materials release Cu ions, which can be harmful to bacterial cells.^30^

In the present work, we prepared TiO_2_ nanotubes (TNTs) by anodic oxidation and then deposited Cu nanoparticles on TNTs using cyclic voltammetry (CV) electrochemical-deposition. By changing the electrodeposition parameters value, different samples were made with different number and size of copper nanoparticles. The purpose in this work is to investigate the bactericidal activity of Cu deposited TNTs. In order to better bactericidal activity investigation, photocatalytic activity of TNTs and Cu deposited TNTs were investigated.

## 2. Experimental

### 2.1. Titanium substrate preparation

Commercially available titanium (Ti) foils (99.5% purity, 1 mm thick) were used as electrode substrates. The foils were cut into 1 × 2 cm^2^ pieces and mechanically polished with sandpaper up to 2500 grit. To remove surface contaminants, the samples were ultrasonically cleaned sequentially in acetone, ethanol, and deionized (DI) water for 15, 10, and 5 minutes, respectively. Subsequently, the Ti foils were chemically etched in a mixture of HNO_3_ and HF (volume ratio 3 : 1) for 3 seconds and then immediately rinsed with DI water.

### 2.2. Fabrication of TiO_2_ nanotube arrays on titanium substrate

TiO_2_ nanotube (TNT) arrays were fabricated on Ti foils using a two-step anodic oxidation process. A two-electrode electrochemical cell was employed, with the Ti foil and a stainless steel 316 plate serving as the anode and cathode, respectively, spaced 3 cm apart. The electrolyte for anodization was prepared by dissolving 0.5 wt% NH_4_F and 2.5 vol% deionized (DI) water in ethylene glycol. A constant voltage of 60 V was applied using a DC power supply (Mastech HY30001E), and the current density was recorded over time with a digital multimeter (Escort 3146A) connected to a computer. The experiment was conducted at room temperature (25 °C).

**Fig. 1:**
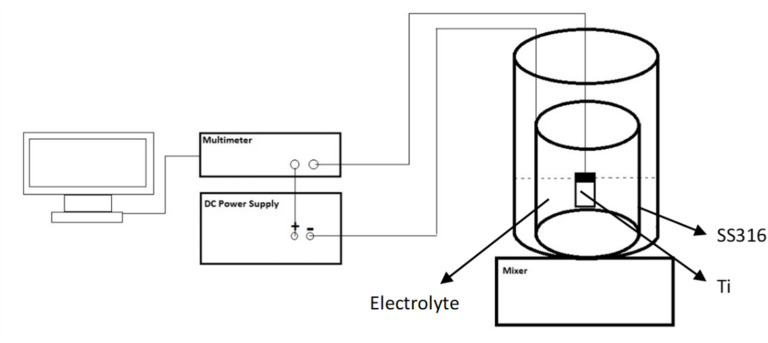
Schematic picture of anodic oxidation of Ti.

The first anodization step lasted 120 minutes. Subsequently, the formed TNT layer was removed by ultrasonication in DI water. The second anodization step was then carried out for 20 minutes. After the second step, the sample was rinsed with DI water and dried in air. Finally, to crystallize the TNTs into the anatase phase, the sample was annealed at 450 °C for 60 minutes. A schematic diagram of the anodic oxidation setup is presented in Fig. 1.

### 2.3. Electrochemical deposition of Cu on TNTs

Copper (Cu) nanoparticles were deposited onto the TiO_2_ nanotube (TNT) arrays *via* cyclic voltammetry (CV) using an Origalys OFG500 potentiostat/galvanostat. A standard three-electrode electrochemical cell was employed, with the TNT sample as the working electrode, an Ag/AgCl electrode as the reference, and a platinum wire as the counter electrode. The deposition electrolyte was prepared by dissolving 0.05 M CuSO_4_·5H_2_O and 0.5 M H_3_BO_3_ in deionized water. The pH of the solution was adjusted to 2 by adding diluted H_2_SO_4_. Cyclic voltammetry was performed over potential ranges from 0 to −1 V (*vs.* Ag/AgCl) for 2, 3, and 5 cycles, using scan rates of 20, 50, and 100 mV s^−1^.

Under strong acidic conditions (pH ≈ 2), Cu^2+^ remains fully solvated and is reduced directly to Cu^0^ without forming hydroxides or oxides. In our experiments, CuSO_4_/H_3_BO_3_ electrolyte was acidified to pH 2 and cycled down to −1 V (*vs.* Ag/AgCl), a potential range where Cu^2+^ → Cu^0^ reduction (*E*° ≈ +0.34 V *vs.* SHE) readily occurs. Low pH suppresses local OH^−^ formation (needed for Cu_2_O/CuO precipitation) and favors proton reduction instead. Consistent with this, recent studies report that low-pH electrolytes strongly disfavour Cu_2_O formation. For example, Mark A. Buckingham *et al.*^31^ note that “low pH… was shown by Siegfried to disfavor cubic Cu_2_O formation”, and that low-pH environments “significantly reduce the voltammetric response of Cu^2+^ ions”, indicating the Cu is being reduced fully to metal rather than to Cu(i) oxide. In short, the acidic medium shifts the electrochemical equilibrium toward Cu metal. This is confirmed by our results: the CV-deposited material shows only metallic-Cu XRD reflections. Fig. 6 of the provided manuscript (TNT-Cu samples) has Cu peaks at 2*θ* = 43.5°, 50.7°, 74.3° ((111), (200), (220) planes), with no Cu_2_O or CuO peaks. In other words, the plating conditions (high [H^+^], sufficient overpotential) drive Cu^2+^ + 2e^−^ → Cu(0) exclusively, preventing oxide formation.

Titania (TiO_2_) is well known to be chemically inert in moderate acids. In fact, TiO_2_ nanotube arrays survive intact in strongly acidic environments. For example, K.-S. Mun *et al.* demonstrated that anodized TiO_2_ nanotube films remain stable across pH 2–12.^32^

**Fig. 2:**
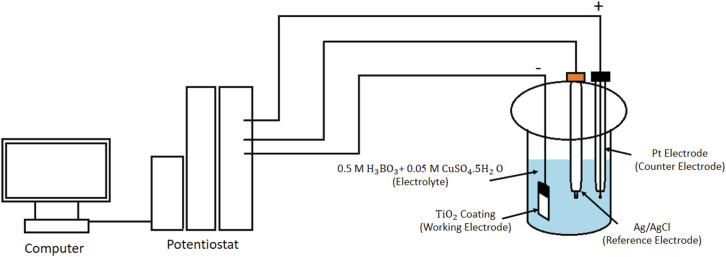
Schematic picture of Cu deposition on TNTs.

The specifications of the prepared samples are listed in Table 1, and a schematic of the Cu deposition setup is illustrated in Fig. 2.

**Table 1:** Sample specifications of TNTs deposited with Cu

Name of sample	Scan rate (mV s^−1^)	Cycle
TNT	—	—
TNT-Cu-3-20	20	3
TNT-Cu-3-50	50	3
TNT-Cu-3-100	100	3
TNT-Cu-2-100	100	2
TNT-Cu-5-100	100	5

### 2.4. Samples characterization

The morphology, size, and distribution of Cu nanoparticles on the TiO_2_ nanotubes (TNTs) were analyzed using field emission scanning electron microscopy (FE-SEM, TESCAN MIRA3 FEG-SEM). The crystalline structures of the TNT and TNT-Cu samples were examined with an X-ray diffractometer (XRD, BRUKER D8 ADVANCE, Germany).

Diffuse reflectance spectroscopy (DRS) was performed on the samples using an SCINCO S-4100 spectrometer over a wavelength range of 300–750 nm. The absorption *versus* wavelength curves obtained from the DRS data were converted to Tauc plots, specifically (*αhν*)^2^*versus* photon energy (*hν*) (eqn (1)–(4)), to calculate the band gap energy of both the pristine and Cu-deposited TNTs.1
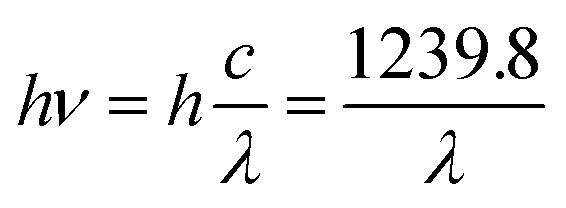
2
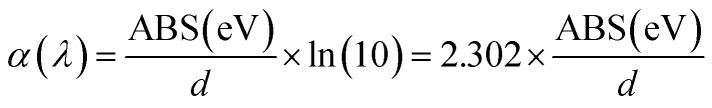

3 ABS (eV) = 27.21 × ABS (A.U)

4
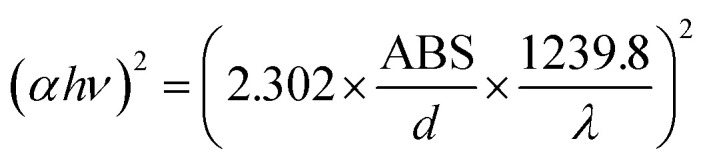


### 2.5. Electrochemical tests

Electrochemical tests, including Mott–Schottky analysis and cyclic voltammetry (CV), were performed using a computer-controlled potentiostat/impedance spectrometer (Origalys OFG500) with a standard three-electrode system. The system consisted of the TNT or TNT-Cu sample as the working electrode, an Ag/AgCl electrode as the reference, and a platinum wire as the counter electrode.

Mott–Schottky analysis was conducted in a 1 M KOH electrolyte. The potential was scanned from −1 V to 0 V (*vs.* Ag/AgCl) at a fixed frequency of 500 Hz. Cyclic voltammetry (CV) was performed in a 0.1 M NaOH electrolyte, with the potential cycled between −1.5 V and +1 V (*vs.* Ag/AgCl) at a scan rate of 5 mV s^−1^.

### 2.6. Bactericidal test

The bactericidal activity of the samples was evaluated against *E. coli* (ATCC 25922), which was purchased from the Iranian Research Organization for Science and Technology. The bacteria were cultured in a nutrient broth (NB) medium to a concentration of 10^6^ cells per mL. A 0.1 mL aliquot of this bacterial suspension was pipetted onto each sample and incubated for 60 minutes at 37 °C.

Following incubation, the samples were exposed to visible light from an LED lamp (8 W, *λ* > 400 nm) for 60 minutes. After irradiation, each sample was gently rinsed with phosphate-buffered saline (PBS) to remove non-adhered bacteria. The adhered bacteria were then detached *via* ultrasonication in 20 mL of PBS. A 100 µL aliquot of the resulting solution was spread onto tryptic soy agar (TSA) plates, which were subsequently incubated at 37 °C for 24 hours.

The bactericidal properties of the samples were quantified by counting the number of bacterial colonies that formed on the TSA plates.

## 3. Results and discussion

### 3.1. TNTs morphology and electrochemical characterization

**Fig. 3:**
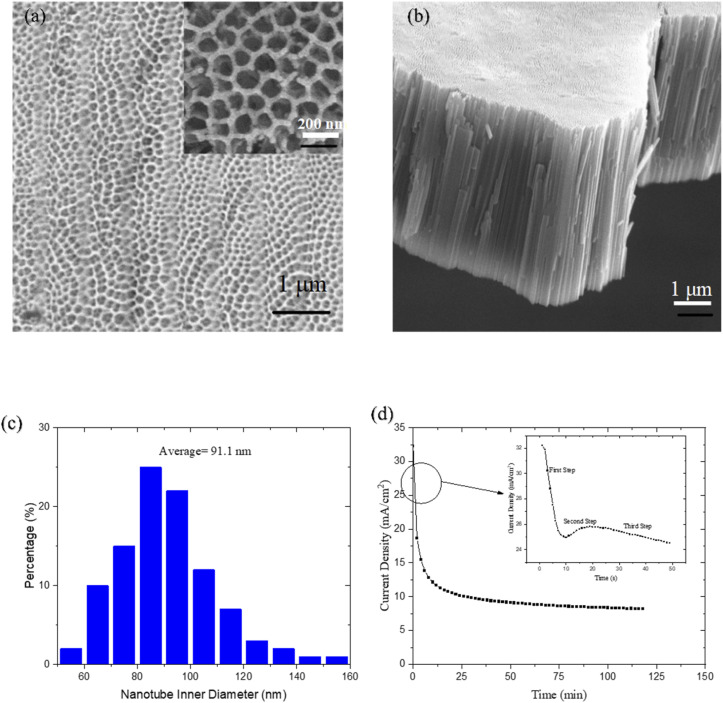
(a) SEM images of top surface and (b) cross section of TNTs, (c) size distribution histogram of inner diameter of TNTs and (d) current density curve *versus* time of first step of anodic oxidation of Ti.

Fig. 3(a) and (b) show SEM images of the top surface and cross-section of the TNTs, respectively, while Fig. 3(c) presents a size distribution histogram of the nanotubes' inner diameter. The two-step anodic oxidation process resulted in the formation of regular nanotubes with a length of approximately 8 µm. The inner diameter of the TNTs ranged from 50 to 160 nm, with an average size of 91.1 nm.

The current density *versus* time curve for the first step of the anodic oxidation is shown in Fig. 3(d). The mechanism of TiO_2_ nanotube formation can be described in three stages. First, a compact TiO_2_ barrier layer forms on the Ti substrate, causing a sharp decrease in current density due to its low conductivity. Second, this compact layer is locally dissolved by fluoride ions, initiating the formation of nanopores. This leads to a slight increase in the current density. In the third stage, these nanopores deepen and spread across the surface, evolving into nanotubes. At the pore bottoms, the TiO_2_ barrier layer becomes thinner, completing the transition to a defined nanotube array.^33^

### 3.2. TNT-Cu morphology and CV curves characterization

**Fig. 4:**
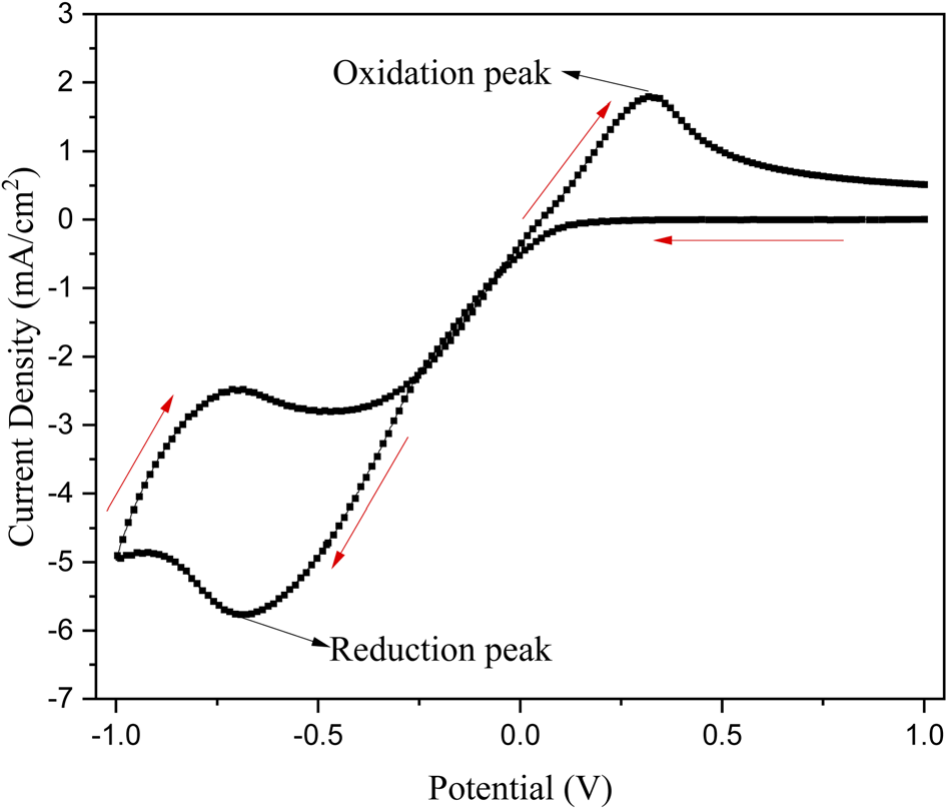
CV curve of Cu deposition on TNTs in potential range from +1 to −1 V at scan rate of 50 mV s^−1^ under 1 cycle.

To investigate the redox processes and confirm the reversibility of copper deposition on the TNTs, cyclic voltammetry (CV) was performed over one cycle in a potential range from +1 V to −1 V at a scan rate of 50 mV s^−1^. The resulting voltammogram is shown in Fig. 4.

The CV profile shows a distinct cathodic (reduction) peak at approximately −701 mV, corresponding to the electrodeposition and growth of Cu nanoparticles. The accompanying anodic (oxidation) peak at approximately +324 mV corresponds to the dissolution of the deposited copper. These observations confirm the reversible nature of the Cu^2+^/Cu redox couple on the TNT substrate. The underlying electrochemical reactions for the reduction and oxidation peaks are proposed as follows:^34^

5 Cu_2_O + 2e^−^ + 2H^+^ → 2Cu + H_2_O (reduction reaction)

6 2Cu^2+^ + 2O^−^ + 2e^−^ → Cu_2_O + H_2_O (oxidation reaction)

Under strongly acidic conditions (pH ≈ 2), Cu^2+^ remains fully solvated and is reduced directly to Cu^0^ without forming hydroxides or oxides. In our experiments the CuSO_4_/H_3_BO_3_ plating bath was acidified to pH 2 and cycled down to −1 V (*vs.* Ag/AgCl), a potential range where Cu^2+^ → Cu^0^ reduction (*E*° ≈ +0.34 V *vs.* SHE) readily occurs. Low pH suppresses local OH^−^ formation (needed for Cu_2_O/CuO precipitation) and favors proton reduction instead. Consistent with this, recent studies report that low-pH electrolytes strongly disfavour Cu_2_O formation.

Titania (TiO_2_) is well known to be chemically inert in moderate acids. In fact, TiO_2_ nanotube arrays survive intact in strongly acidic environments. For example, K.-S. Mun *et al.* demonstrated that anodized TiO_2_ nanotube films remain stable across pH 2–12.^32^

**Fig. 5:**
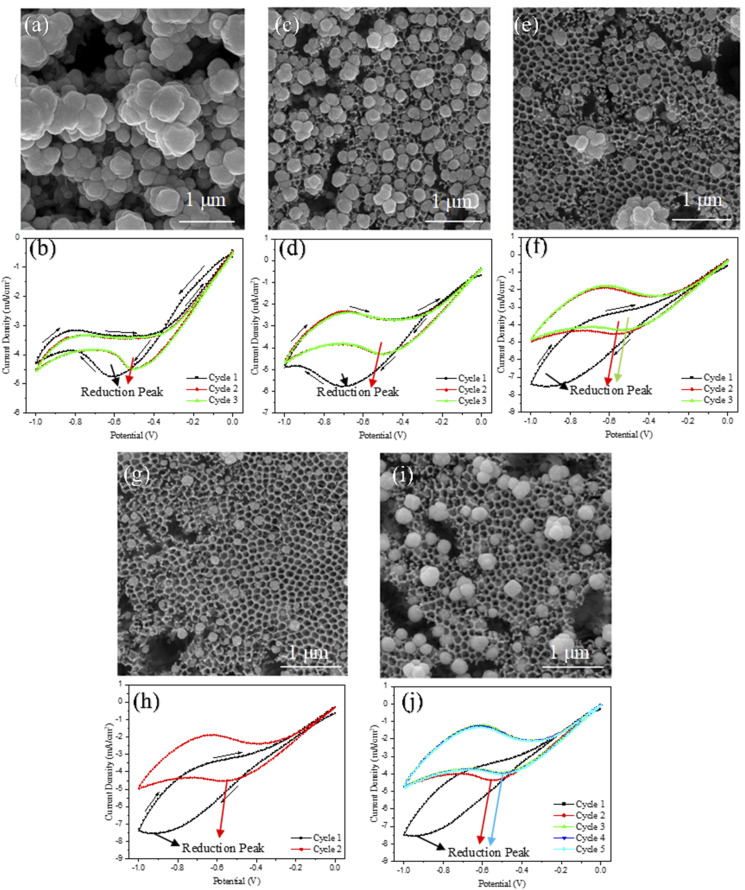
FE-SEM images of (a) TNT-Cu-3-20, (c) TNT-Cu-3-50, (e) TNT-Cu-3-100, (g) TNT-Cu-2-100 and (i) TNT-Cu-5-100 and CV deposition curves of (b) TNT-Cu-3-20, (d) TNT-Cu-3-50, (f) TNT-Cu-3-100, (h) TNT-Cu-2-100 and (j) TNT-Cu-5-100.

Fig. 5 shows FE-SEM images of Cu-deposited TNTs and the corresponding cyclic voltammetry (CV) curves under different scan rates and cycle numbers. The Cu nanoparticles deposited on the TNTs exhibit a quasi-spherical morphology, which is influenced by the presence of boric acid in the electrolyte.

When the number of cycles was held constant at 3, an increase in the scan rate resulted in smaller Cu nanoparticles with less surface coverage. The CV curves provide insight into this behavior. The intersection between the forward and return sweeps indicates the nucleation of Cu nanoparticles. This nucleation loop is clearly visible in the CV curve at 20 mV s^−1^ but is absent at 100 mV s^−1^. At higher scan rates, it is proposed that a greater number of nucleation sites are occupied by hydrogen ions (H^+^), which compete with Cu^2+^ ions. This competition leads to a decrease in the number of Cu nanoparticles deposited as the scan rate increases.^35^ The growth of Cu nanoparticles occurs during the reduction peak. As the scan rate increases, the duration of the potential sweep through the reduction region decreases. This shorter timeframe limits the growth of the Cu nanoparticles compared to depositions performed at lower scan rates. Consequently, higher scan rates result in both a smaller size and a lower population density of Cu nanoparticles.

Conversely, when the number of deposition cycles was increased at a constant scan rate (100 mV s^−1^), the existing Cu nanoparticles grew larger, primarily through coalescence. This phenomenon is attributed to the higher electrical conductivity of copper compared to the TiO_2_ substrate and the limited availability of favorable nucleation sites. With each subsequent cycle, deposition preferentially occurs onto the existing metallic Cu nanoparticles rather than forming new nuclei on the less conductive TiO_2_ surface.

In the other words, more cycles on an initially non-conductive surface reduce the density of new nucleation sites (many have been occupied) and drive lateral growth of existing particles. This island-growth (Volmer–Weber) behavior arises because Cu–Cu deposition is easier (lower overpotential) on Cu than Cu–TiO_2_ deposition. The formation of conductive Cu islands also alters the local electrochemistry. Electron transfer and reduction reactions occur more readily at those islands, so edges and defects on existing Cu accumulate even more current. In Corso *et al.*'s^36^ work, excessive Cu (many CV cycles) even “hinders electron transfer” in mass-transport limited situations, effectively slowing nucleation of new particles and favoring growth of large clusters. Seed-site growth: consistent with this, prior studies report that once metal clusters form, they become preferred growth centers. Corso *et al.*^36^ observed that with increasing CV cycles, “the crystallites already formed act as nucleation centers” for new Cu, leading to larger merged clusters. Their SEMs show 2 CV cycles give small, isolated Cu particles, while 5–15 cycles yield extensive, flower-like Cu coverage (the carbon surface is almost fully covered and Cu structures merge). In other words, more cycles on an initially non-conductive surface reduce the density of new nucleation sites (many have been occupied) and drive lateral growth of existing particles. This island-growth (Volmer–Weber) behavior arises because Cu–Cu deposition is easier (lower overpotential) on Cu than Cu–TiO_2_ deposition. Local current focusing and particle coalescence: the formation of conductive Cu islands also alters the local electrochemistry. Electron transfer and reduction reactions occur more readily at those islands, so edges and defects on existing Cu accumulate even more current. In Corso *et al.*'s^36^ work, excessive Cu (many CV cycles) even “hinders electron transfer” in mass-transport limited situations, effectively slowing nucleation of new particles and favoring growth of large clusters. In our case, after a few CV cycles the FESEM in Fig. 5 shows Cu NPs coalescing – small particles become joined into larger aggregates. This matches the literature: *e.g.* after 2 CV cycles only sparse Cu spots appear, but by 5+ cycles the coverage is nearly uniform and clusters have merged. In short, once a partial Cu film exists, further deposition “fills in” and thickens those islands instead of creating many new nuclei. CV evidence of increased conductivity: the CV data reflect this mechanism. The manuscript notes the first-cycle reduction peak is at lower current (and slightly more negative potential) than in later cycles. This exactly indicates higher electrode impedance initially: with only TiO_2_ and a few Cu nuclei, the effective surface conductivity is low, so less current flows for a given overpotential. After the first cycle, added Cu lowers the film resistance, so subsequent cycles show larger cathodic current densities and a positive shift of the peak potential. In other words, the increasing Cu coverage improves charge transfer, consistent with the “higher conductivity of Cu than TiO_2_” explanation. Thus, the observed CV trends (growing peak currents at fixed scan rate) and the FESEM (particle coalescence with cycle number) both support the conductivity-driven growth model.

The potential and current density values for the Cu reduction peaks during CV deposition on TNTs are summarized in Table 2 for various scan rates and cycle numbers. Analysis of the CV curves and the data in Table 2 indicates that both the reduction potential and current density in the first cycle are lower than in subsequent cycles. This can be attributed to the lower effective conductivity of the electrode during the initial cycle, as the bare TNT substrate is less conductive than after it has been decorated with metallic copper nanoparticles.^37^ As the scan rate increased, the reduction peak shifted to higher potentials and the peak current density increased. This behavior is characteristic of a quasi-reversible or irreversible electrochemical system. In a perfectly reversible system, electron transfer is fast enough to maintain Nernstian equilibrium at the electrode surface at all times. The peak current increases with the square root of the scan rate, but the peak potential remains constant.

**Table 2:** Potential and current density of reduction peaks of Cu CV deposition for each cycles and scan rate

Cu CV deposition scan rate (mV s^−1^)	Cycle number	Potential of reduction peak (mV)	Current density of reduction peak (mA cm^−2^)
20	1	−614	−4.75
	2	−501	−4.48
	3	−500	−4.50
50	1	−701	−5.77
	2	−511	−4.28
	3	−511	−4.28
100	1	−927	−7.55
	2	−570	−4.53
	3	−540	−4.31
	4	−540	−4.32
	5	−540	−4.33

However, as the scan rate increases in a system with slower electron transfer kinetics (quasi-reversible or irreversible), the system deviates from equilibrium. The electron transfer rate becomes insufficient to maintain the Nernstian condition, requiring a higher overpotential (a more negative potential for reduction) to drive the reaction at the increased mass transfer rate. This manifests as a positive shift in the reduction peak potential. Concurrently, the peak current density increases due to the steeper concentration gradient established by the faster scan rate.^38^

**Fig. 6:**
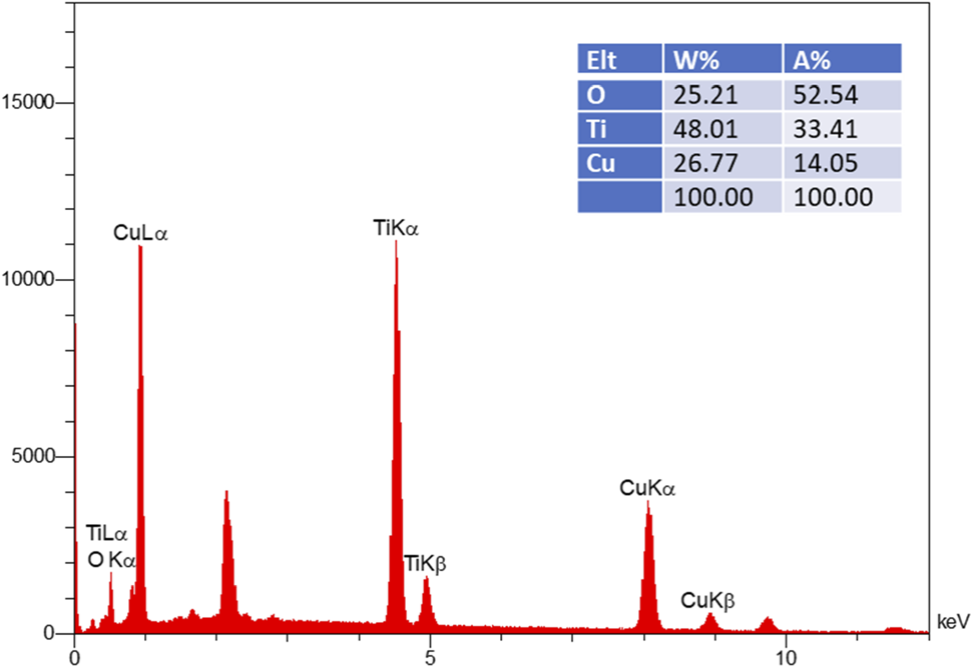
Energy-dispersive X-ray (EDX) spectrum of the TNT-Cu.

EDX analysis (Fig. 6) confirms the presence of copper on the TNT samples. This finding, which aligns with the FESEM results, verifies that the deposited particles are copper nanoparticles. The presence of these nanoparticles is directly responsible for the observed antibacterial activity.

### 3.3. XRD patterns characterization

**Fig. 7:**
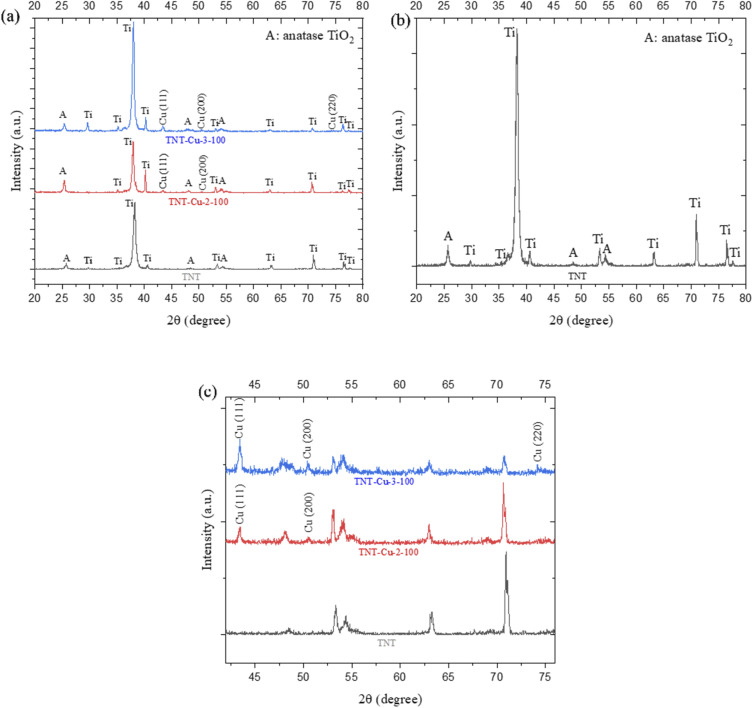
(a) XRD patterns of TNT, TNT-Cu-2-100 and TNT-Cu-3-100, (b) magnified XRD pattern of TNT and (c) magnifies XRD patterns of TNT, TNT-Cu-2-100 and TNT-Cu-3-100.

Fig. 7 presents the XRD patterns of pristine TNT, TNT-Cu-2-100, and TNT-Cu-3-100, recorded over a 2*θ* range of 20° to 80°. Peaks corresponding to metallic Ti and anatase TiO_2_ are present in all three samples. The anatase TiO_2_ peaks are observed at 2*θ* = 25.7°, 48.1°, and 54.4°, confirming that the annealing process at 450 °C for 60 minutes successfully crystallized the amorphous TiO_2_ into the anatase phase. The Ti substrate peaks are observed at 2*θ* = 35.2°, 38.2°, 40.6°, 53.3°, 63.1°, 70.9°, 76.5°, and 77.6°. These assignments agree with standard reference data: the ICDD (JCPDS) powder diffraction file PDF #21-1272 (formerly JCPDS Card No. 21-1272) lists anatase TiO_2_ with those same peak positions and indices.

The XRD peaks in our anodized TiO_2_ nanotube sample match well-known anatase reflections. For example, 2*θ* ≃ 25.3°, 37.8°, 48.0°, 53.9°, 55.0°, and 62.7° correspond respectively to the (101), (004), (200), (105), (211), and (204) planes of tetragonal anatase TiO_2_. These assignments agree with standard reference data: the ICDD (JCPDS) powder diffraction file PDF #21-1272 (formerly JCPDS Card No. 21-1272) lists anatase TiO_2_ with those same peak positions and indices. (Older literature sometimes cites JCPDS 78-2486 for anatase, but the PDF #21-1272 is the current standard for anatase TiO_2_.) Thus, all observed peaks in the 25–63° range index unambiguously to anatase crystal planes.^39^

In addition to the anatase peaks, our XRD shows weaker reflections at ∼35–40° and ∼53°, which match metallic Ti (α-Ti). For example, prominent Ti peaks appear near 35.1°, 38.4°, 40.2° and 53° (corresponding to Ti (100), (002), (101), (102) planes, respectively). These are well-known features of the Ti metal substrate: one XRD study of anodized TiO_2_ nanotubes reports four Ti-metal reflections at 35.1°, 38.4°, 40.2° and 53° (JCPDS #44-1294 for α-Ti) appearing under the nanotube layer. Crucially, the literature emphasizes that such Ti peaks arise purely from X-ray penetration through the thin oxide film into the Ti foil, not from any chemical reduction of TiO_2_. For example, some papers note that these peaks “correspond to the titanium substrate” beneath the TiO_2_ layer.^40^ Similarly, Thierry Djenizian *et al.* explicitly state “The Ti peaks observed on the XRD analysis are [from] the Ti substrate due to the direct growth of TiO_2_ nanotubes on the Ti grid” also Eagambaram Murugan *et al.* In short, detection of α-Ti reflections is expected when measuring thin TiO_2_ coatings on Ti, and does not imply that the anatase has decomposed or been reduced (the TiO_2_ remains intact).^41,42^

A comparison of the patterns in Fig. 7(c) reveals that the intensity of the Cu peaks in the TNT-Cu-2-100 sample is lower than in the TNT-Cu-3-100 sample. This is consistent with lower surface coverage and a smaller quantity of Cu nanoparticles on the TNT-Cu-2-100 sample, which underwent fewer deposition cycles. For the TNT-Cu-3-100 sample, distinct Cu peaks are observed, which can be indexed to the (111) plane at 43.5°, the (200) plane at 50.7°, and the (220) plane at 74.3°. In contrast, the Cu (220) plane is not detectable in the XRD pattern of the TNT-Cu-2-100 sample, further supporting its lower Cu content.

Cheshideh *et al.*^35^ reports about Ni deposited TNTs that when the scan rate increases from 20 to 50 mV s^−1^ the particle size is smaller than the critical size solely forming (111) direction peak. Furthermore, for the TNT-Cu-2-100 sample, the Cu nanoparticles may be smaller than the critical size required to form a detectable diffraction peak for the (220) plane. The higher intensity of the (111) peak compared to other Cu peaks indicates a preferred crystal growth orientation, suggesting that a majority of the Cu nanoparticles are oriented with their (111) lattice planes parallel to the substrate.^43^

### 3.4. DRS curves characterization

**Fig. 8:**
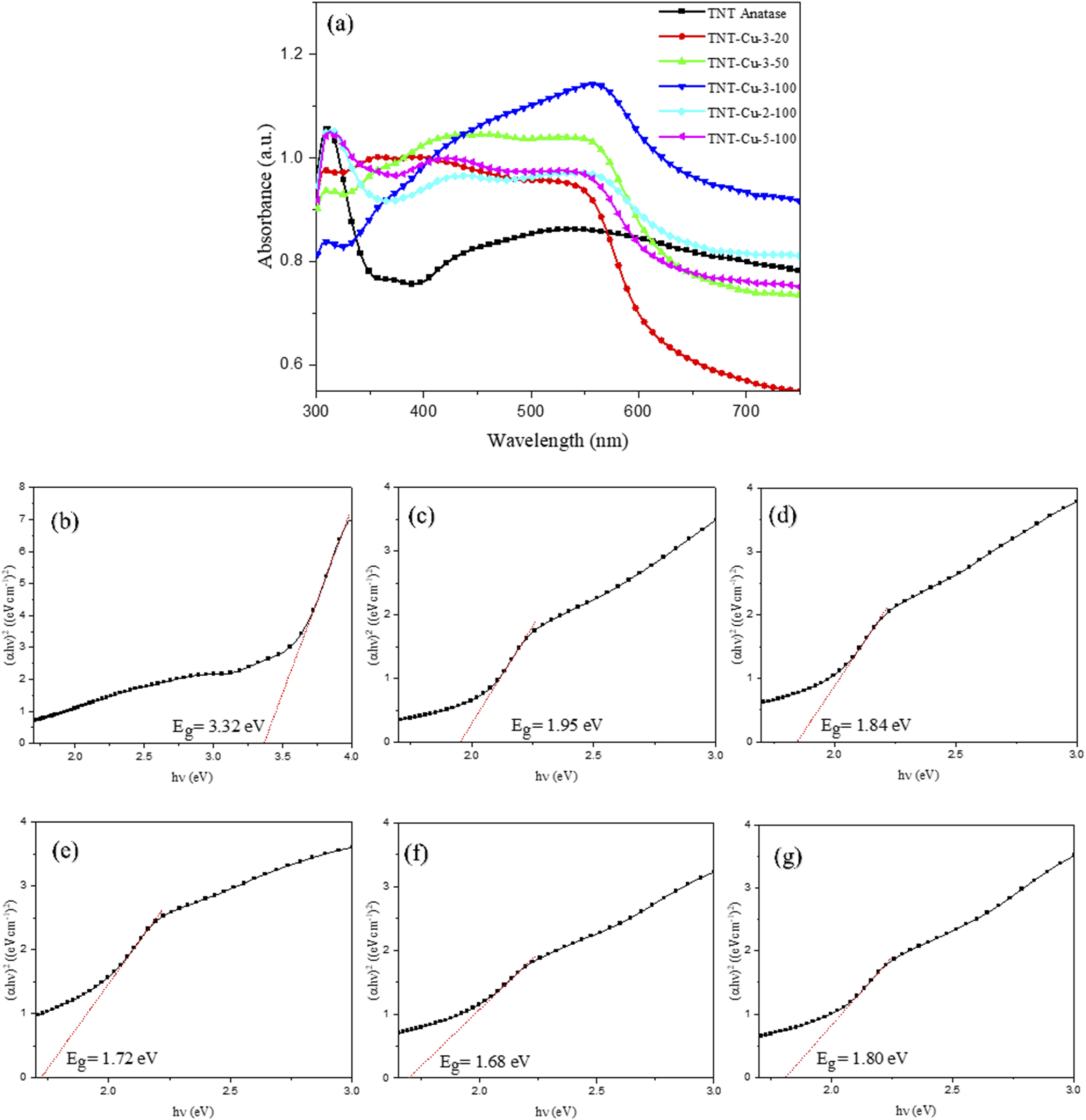
(a) DRS plots of TNTs and Cu deposited TNTs and plots of (*αhν*)^2^*versus* the photon energy (*hν*) of (b) TNT, (c) TNT-Cu-3-20, (d) TNT-Cu-3-50, (e) TNT-Cu-3-100, (f) TNT-Cu-2-100 and (g) TNT-Cu-5-100.

Fig. 8(a) presents the diffuse reflectance spectroscopy (DRS) curves for the pristine TNTs and Cu-deposited TNTs over a wavelength range of 300–750 nm. The deposition of Cu nanoparticles on the TNTs resulted in a clear redshift of the absorption edge into the visible light region. Specifically, the TNT-Cu-2-100 and TNT-Cu-3-100 samples exhibited significantly higher absorption in the visible spectrum compared to the pristine TNTs. In contrast, samples TNT-Cu-3-20, TNT-Cu-3-50, and TNT-Cu-5-100 showed lower absorption than the TNTs at wavelengths above 570 nm, 610 nm, and 595 nm, respectively. The band gap energies were determined by converting the DRS data to Tauc plots, specifically (*αhν*)^2^*versus* photon energy (*hν*), as shown in Fig. 8(b–g). The calculated band gap energies were 3.32 eV for pristine TNTs, and 1.95, 1.84, 1.72, 1.68, and 1.80 eV for TNT-Cu-3-20, TNT-Cu-3-50, TNT-Cu-3-100, TNT-Cu-2-100, and TNT-Cu-5-100, respectively. Among the modified samples, TNT-Cu-2-100 possessed the narrowest band gap.

Mathew *et al.*,^23^ Miao *et al.*^44^ and Yadav *et al.*^45^ also reported that addition of Cu on TiO_2_ nanostructure material, improved the light absorption properties under the visible light irradiation. This indicates that band gap energy reducing and absorption increasing under visible light, requires the addition of metal ions on TiO_2_.^46^ In order to further reduce of the band gap energy of TNTs by Cu deposition, better interaction between TiO_2_ and Cu is required.^47^ One critical factor for achieving strong electronic interaction between TiO_2_ and Cu is the uniform dispersion of Cu nanoparticles on the TNTs. As previously demonstrated by the FE-SEM analysis, the TNT-Cu-2-100 sample exhibited the most homogeneous distribution of Cu nanoparticles. This optimal dispersion is a key reason why this sample achieved the lowest band gap energy among all the Cu-deposited TNTs.

The reduced band gap energy lowers the energy required to excite electrons from the valence band to the conduction band. Consequently, electron transfer can be initiated by lower-energy photons within the visible light spectrum. This enhanced photoactivity under visible light leads to a higher generation of charge carriers, thereby increasing the electron donor density available for photocatalytic reactions.

### 3.5. Mott–Schottky curves characterization

**Fig. 9:**
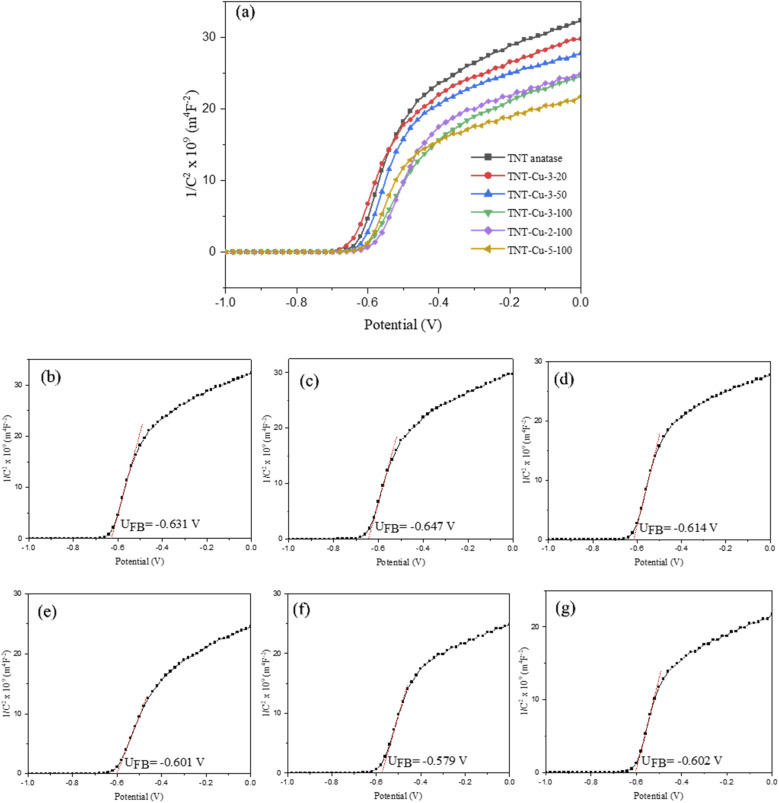
(a) Mott–Schottky plots of TNTs and Cu deposited TNTs together and Mott–Schottky plots of (b) TNT, (c) TNT-Cu-3-20, (d) TNT-Cu-3-50, (e) TNT-Cu-3-100, (f) TNT-Cu-2-100 and (g) TNT-Cu-5-100 separated.

The Mott–Schottky test was carried out to investigate semiconductor type and calculate Flat band potential (UFB).^48^Fig. 9 shows the Mott–Schottky plots for the pristine TNTs and Cu-deposited TNTs, measured at a frequency of 500 Hz under visible light irradiation. The positive slopes of the curves for all samples confirm their behavior as n-type semiconductors. The flat-band potential (UFB) for each sample was determined by extrapolating the linear portion of the Mott–Schottky plot to 1/*C*^2^ = 0. The calculated UFB values were −0.631 V for TNT, and −0.647, −0.614, −0.601, −0.579, and −0.602 V for TNT-Cu-3-20, TNT-Cu-3-50, TNT-Cu-3-100, TNT-Cu-2-100, and TNT-Cu-5-100, respectively. Among these, TNT-Cu-2-100 exhibited the most positive (or least negative) flat-band potential. A shift of the UFB in the positive direction indicates an upward shift in the Fermi level of the semiconductor.^49^ This upward shift in the Fermi level facilitates electron excitation from the valence band to the conduction band. The resulting increase in the population of charge carriers enhances the rate of photocatalytic reactions.

### 3.6. Bactericidal activity

**Fig. 10:**
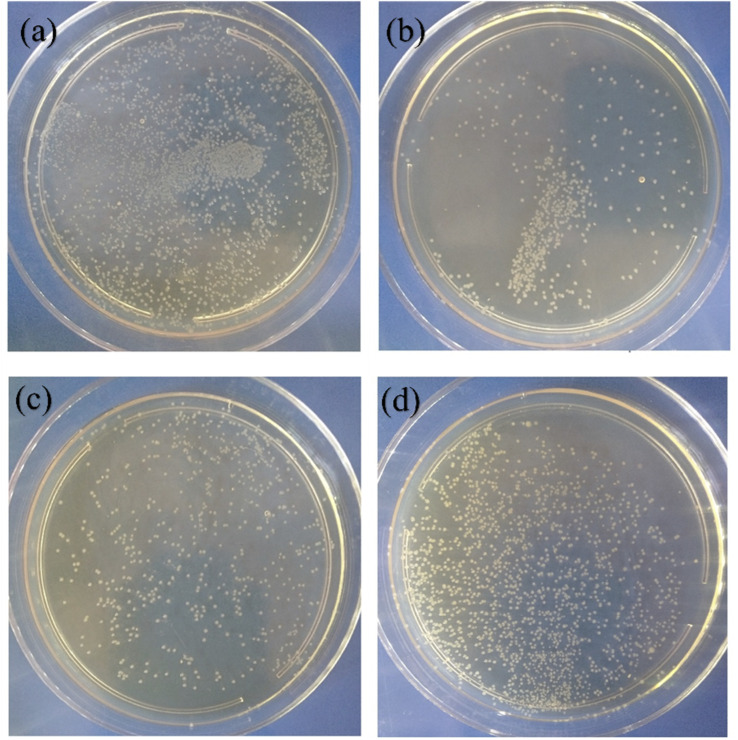
TSA plates of bactericidal activity of (a) TNT, (b) TNT-Cu-2-100 and (c) TNT-Cu-3-100 under visible light and (d) TNT-Cu-2-100 in dark.

**Fig. 11:**
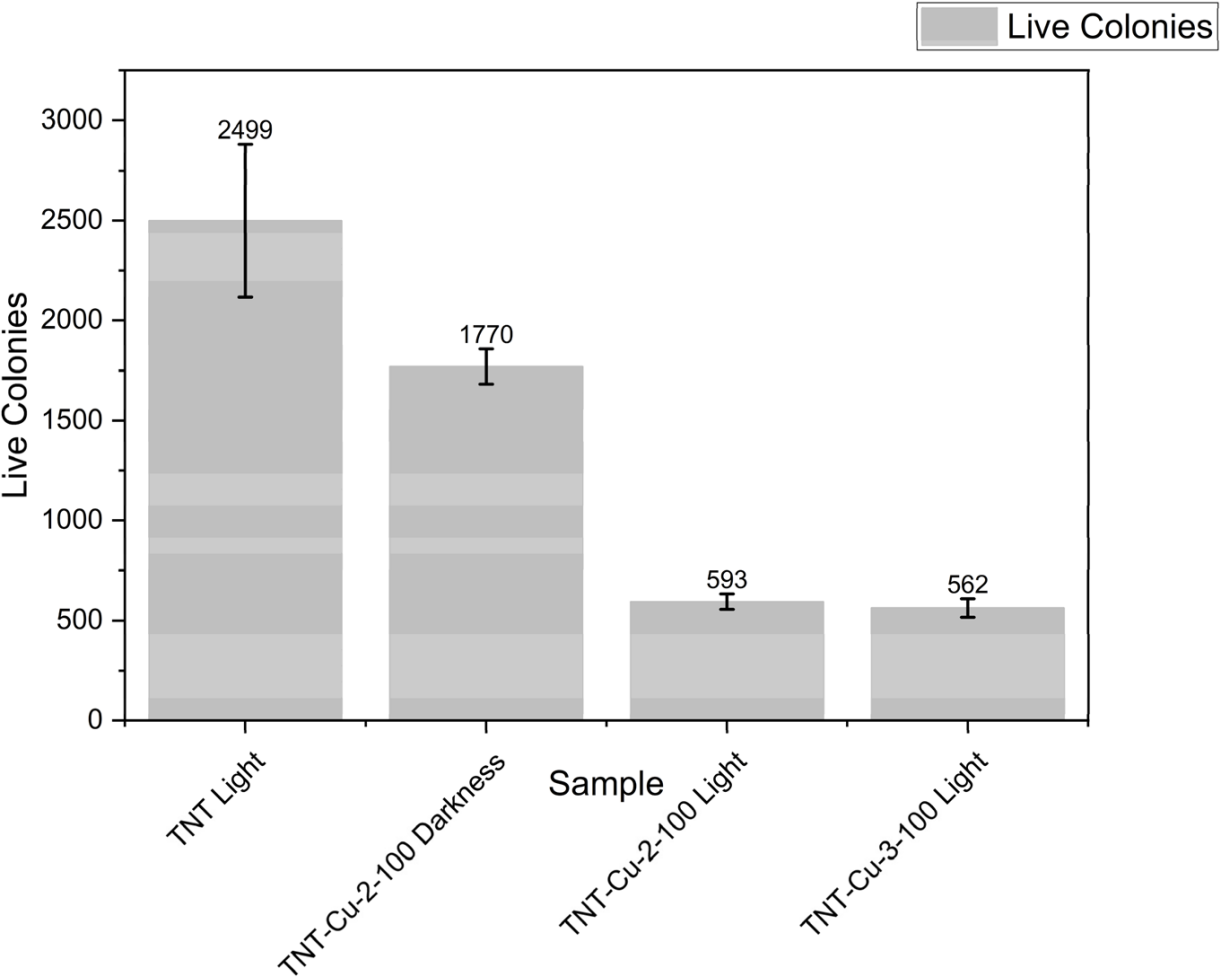
Column charts of counting live bacterial colonies on TSA plates related to bactericidal activity of TNT, TNT-Cu-2-100 and TNT-Cu-3-100 under visible light and TNT-Cu-2-100 in dark.

The bactericidal activity was evaluated for TNT, TNT-Cu-2-100, and TNT-Cu-3-100 under visible light irradiation, and for TNT-Cu-2-100 in the dark. Fig. 10 shows the tryptic soy agar (TSA) plates from these tests, where the live bacterial colonies are visible. A lower number of colonies on a plate indicates higher bactericidal activity. The quantitative colony counts are presented in the column chart in Fig. 11. The number of live colonies for TNT, TNT-Cu-2-100, and TNT-Cu-3-100 under visible light were 2560, 530, and 570, respectively. This corresponds to a bactericidal activity that is 79.3% and 77.7% higher for TNT-Cu-2-100 and TNT-Cu-3-100, respectively, compared to pristine TNTs under visible light. This confirms that Cu deposition significantly enhances the bactericidal performance under visible light. Furthermore, TNT-Cu-2-100 also exhibited a notable bactericidal effect in the dark, with a colony count of 1840. This dark activity was 28.2% higher than the activity of pristine TNTs under visible light, demonstrating that the composite possesses intrinsic antibacterial properties, which are further amplified by visible light irradiation.

The bactericidal mechanism of the Cu-deposited TNTs can be attributed to two primary factors: their enhanced visible-light photocatalytic activity and the inherent antimicrobial properties of released copper ions.^50,51^ As demonstrated, the deposition of Cu on TNTs caused a redshift of the absorption edge and a reduction in the band gap energy. This modification sensitizes the material to visible light, enabling photocatalytic reactions under visible light irradiation.

**Fig. 12:**
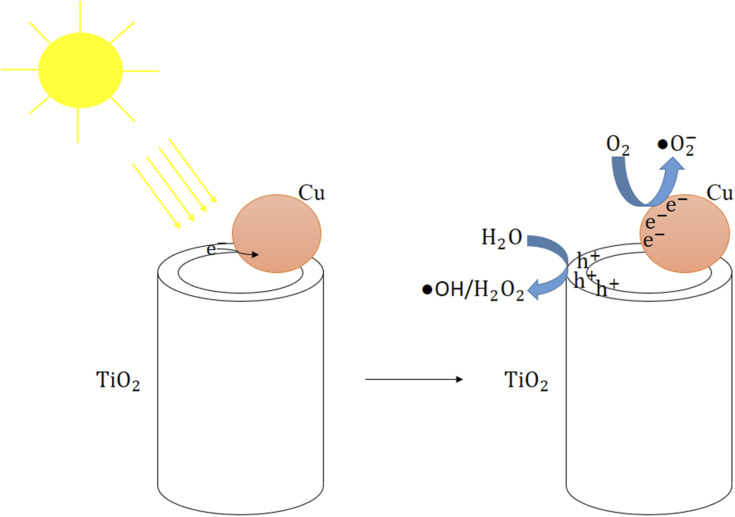
The bactericidal mechanism schematic of Cu deposited TNTs under visible light irradiation.

As illustrated in Fig. 12 and reactions (7)–(14), upon visible light irradiation, electrons are excited from the valence band of TiO_2_ and are subsequently transferred to Cu nanoparticles. This process leads to the generation of electron–hole pairs in both materials. These charge carriers then react with surrounding molecules (*e.g.*, H_2_O, O_2_) to generate reactive oxygen species (ROS) such as OH, H_2_O_2_, and O_2_^−^.^44,52^ The generated ROS are highly reactive and can attack bacterial cells, leading to the degradation of the cell wall and damage to intracellular components such as DNA. The primary mechanisms of damage include the abstraction of hydrogen atoms from organic molecules and the disruption of essential protein structures, ultimately causing cell death.^53^ As seen, TNT-Cu-2-100 showed slightly bactericidal activity in dark. This indicates antimicrobial properties of Cu ions.

7 Cu/TiO_2_ + *hv* → e_CB_^−^(Cu) + h_VB_^+^(TiO_2_)

8 e_CB_^−^ + O_2_ → ˙O_2_^−^

9 h_VB_^+^ + OH^−^ → ˙OH

10 h_VB_^+^ + H_2_O → ˙OH + H^+^

11
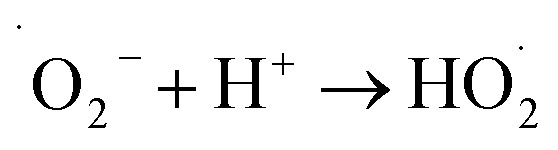
12
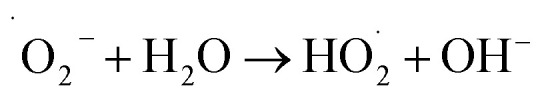
13
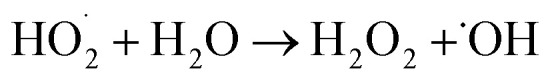

14 H_2_O_2_ → 2˙OH

As previously stated, the TNT-Cu-2-100 sample exhibited superior bactericidal activity compared to TNT-Cu-3-100. This enhanced performance can be explained by its more effective photocatalytic mechanism. The lower band gap energy of TNT-Cu-2-100 facilitates a higher rate of electron excitation from the valence band to the conduction band under visible light. This results in a greater generation of electron–hole pairs, which intensifies the photocatalytic reactions and leads to more efficient disinfection.

Furthermore, the intrinsic antimicrobial properties of copper metal contribute significantly to the overall effect. The synergistic combination of enhanced visible-light photocatalysis and the inherent bactericidal activity of copper nanoparticles is therefore responsible for the high antibacterial performance of the TNT-Cu.

## 4. Conclusion

In this study, the bactericidal activity of copper-deposited anatase TiO_2_ nanotubes (TNTs) was successfully evaluated under visible light irradiation. FE-SEM analysis confirmed that the TNT-Cu-2-100 sample possessed the most uniform dispersion of Cu nanoparticles. Optical characterization *via* DRS revealed that Cu deposition induces a redshift in the absorption edge and significantly reduces the band gap energy. Specifically, the optimal sample (TNT-Cu-2-100) exhibited a band gap of 1.68 eV, a substantial reduction from the 3.32 eV of pristine TNTs.

Electrochemical Mott–Schottky analysis further demonstrated that TNT-Cu-2-100 has the most positive flat band potential (−0.579 V) compared to pristine TNTs (−0.631 V), indicating a raised Fermi level favorable for photocatalysis. Consequently, this sample demonstrated the highest bactericidal performance, showing a 79.3% enhancement in efficacy under visible light compared to unmodified TNTs. While a significant bactericidal effect was also observed in the dark—attributed to the intrinsic property of copper—the activity was substantially amplified under visible light irradiation, confirming the crucial role of the enhanced photocatalytic mechanism.

## Conflicts of interest

The authors declare that they have no known competing financial interests or personal relationships that could have appeared to influence the work reported in this paper.

## Data Availability

The authors confirm that the data supporting the findings of this study are available within the article. Data will be available on request.
